# COVID-19 Coexisting With the Human Immunodeficiency Virus: A Case Report

**DOI:** 10.7759/cureus.11007

**Published:** 2020-10-17

**Authors:** Elizabeth Gamboa, Melanie Duran, Joseph C Gathe, Salim Surani, Joseph Varon

**Affiliations:** 1 Medicine, Universidad Xochicalco, Ensenada, MEX; 2 Internal Medicine, United Memorial Medical Center, Houston, USA; 3 Infectious Disease, United Memorial Medical Center, Houston, USA; 4 Internal Medicine, Corpus Christi Medical Center, Corpus Christi, USA; 5 Internal Medicine, University of North Texas, Dallas, USA; 6 Critical Care, United Memorial Medical Center, Houston, USA

**Keywords:** coronavirus disease 2019, covid-19, sars-cov-2, human immunodeficiency virus, cd4+ count

## Abstract

The newly discovered severe acute respiratory syndrome coronavirus 2 (SARS-CoV-2) has impacted the world dramatically, forcing the medical community to quickly and effectively find ways to manage coronavirus disease 2019 (COVID-19). The COVID-19 pandemic has shown many similarities to the human immunodeficiency virus pandemic in 1981, from the fear of treating patients for a virus we have little knowledge of, to analyzing how the levels of CD4+ are affected in both diseases. Declining numbers of CD4+ levels are classically seen with HIV patients, however, given the immune response of our bodies, these levels have also been seen to decrease during an active COVID-19 infection. Besides, there is speculation that people living with HIV are at a higher risk for mortality if infected with SARS-CoV-2. Therefore, the interaction of these two viruses can create a syndemic culture, and thus, the need to monitor and treat patients with human immunodeficiency virus and COVID-19 cautiously.

## Introduction

At the end of December 2019, the severe acute respiratory syndrome coronavirus 2 (SARS-CoV-2) was identified in Wuhan City, Hubei Province, China, as the cause of the outbreak of pneumonia of unknown etiology [1]. As of September 13, 2020, there have been 28 million cases confirmed and 917,417 deaths around the world [2]. Immunological changes are a hallmark of the infection, yet we do not have a clear understanding of the immune process. The total lymphocyte count and absolute CD4+ helper lymphocytes in peripheral blood can decrease significantly [3]. This is similar to the pathogenesis of the human immunodeficiency virus (HIV), which is manifested by a decline in T-lymphocytes, a decrease in total CD4+ count, and will subsequently cause immunosuppression [4]. A co-infection of HIV and coronavirus disease 2019 (COVID-19) can have a significant impact on the immune system of an individual. Little is known about the effects on patients with COVID-19 simultaneously infected with HIV. In this article, we describe one such case.

## Case presentation

**Figure 1:**
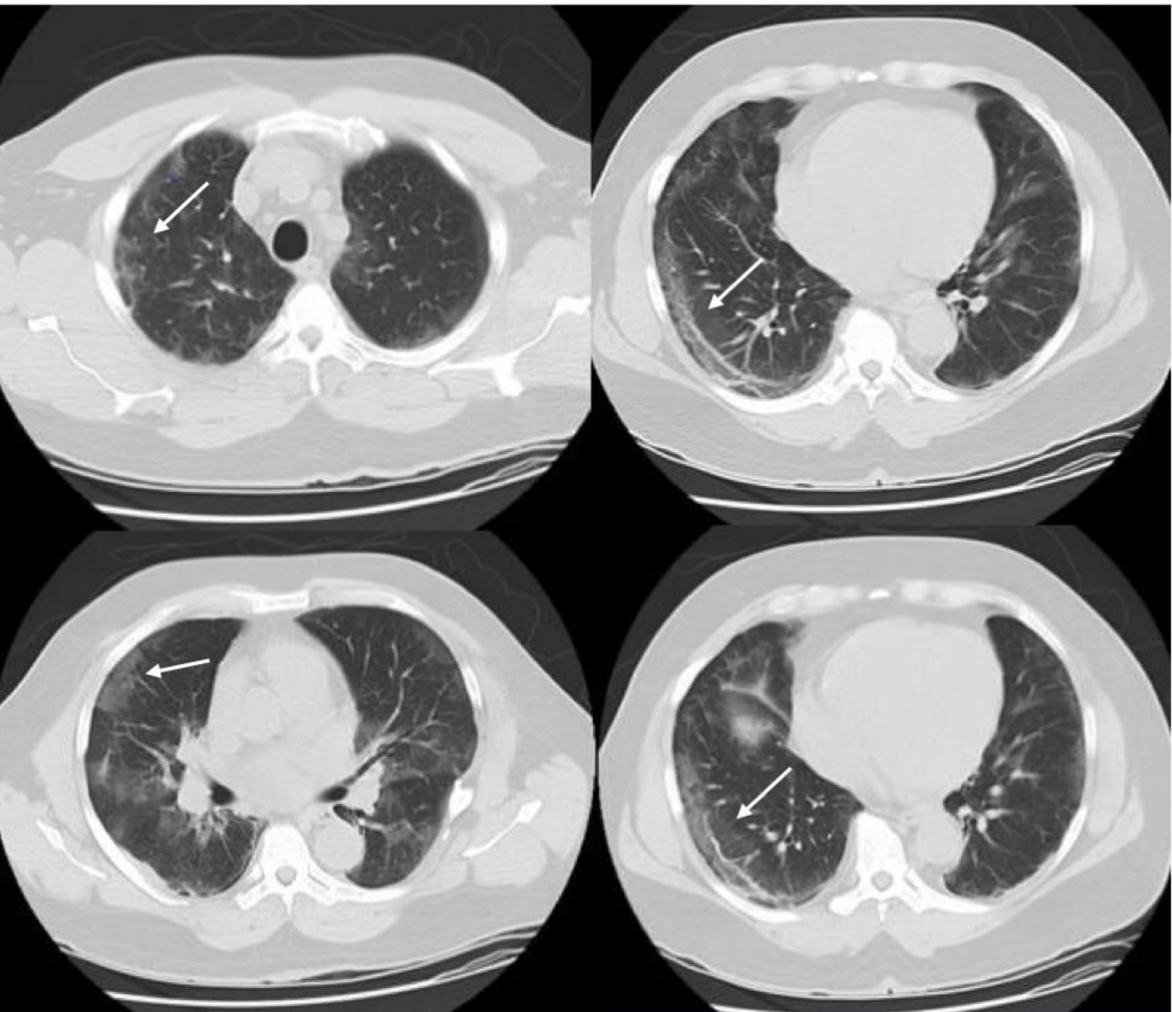
Non-contrast CT scan of the chest Day 1 of hospital admission. Non-contrast CT image of the chest showing multiple, bilateral ground-glass opacities (white arrows) in both upper and lower lobes with a peripheral distribution.

A 59-year-old African American gentleman presented to our facility with progressive dry cough, worsening shortness of breath, loss of appetite, nausea, diarrhea, anuria and generalized weakness. The patient had a past medical history of chronic hypertension, end-stage renal disease receiving renal replacement therapy, and HIV diagnosed in 1994 treated with oral antiretroviral therapy. He had recently tested positive for COVID-19, by a reverse transcription polymerase chain reaction (RT-PCR) nasopharyngeal swab sample. A computed tomography (CT) of the chest depicted bilateral ground-glass opacities in both upper and lower lobes (Figure 1). Upon admission, his blood pressure was 140/80 mmHg, heart rate 99 beats/min, respiratory rate 26/min, temperature 39.4°C and SaO₂ 94% while breathing on 2 liters/minute by nasal cannula. Given the patient’s comorbidities, persistent fever, chest CT findings, and positive RT-PCR for COVID-19, the patient was admitted and placed under strict isolation. He initiated the MATH+ treatment protocol including methylprednisolone, ascorbic acid, thiamine, heparin, atorvastatin, zinc, vitamin D3, famotidine, magnesium, melatonin, and azithromycin [5]. His current antihypertensive and antiretroviral therapy home medications were also continued upon hospital admission. 

**Figure 2:**
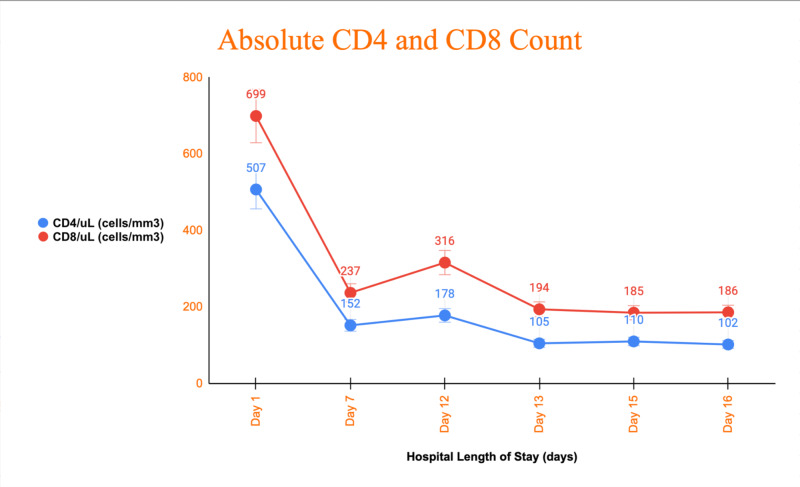
Absolute CD4 and CD8 count during hospital stay This graph demonstrates the decline in absolute CD4+ and CD8+ levels, expressed in cells/mm³, throughout the patient’s hospital stay.

The patient had an undetectable HIV viral load and a baseline CD4+ count of 865 cells/mm^3^ during his previous follow up visit with his infectious disease specialist one month prior. On admission, however, he had a CD4+ count of 507 cells/mm^3^. Daily CD4+ and CD8+ blood tests were drawn to manage the patient accordingly. On day two of hospitalization, the patient’s oxygen saturation and respiratory status began to rapidly deteriorate requiring oxygen supplementation with high flow nasal cannula at 20 liters/min and fraction of inspired oxygen (FiO_2_) 60%. Throughout the course of his stay, the patient’s absolute CD4+ and CD8+ lymphocyte count showed a progressive decline (Figure 2). He was started on Bactrim and Diflucan to prevent opportunistic disease. Over the next few days, the patient’s clinical presentation improved with supplemental oxygen and continuous administration of the MATH+ treatment protocol. He was discharged home after a two-week hospital stay and a negative RT-PCR test for SARS-CoV-2. His outpatient follow-up visit one week later reported that the patient’s CD4+ count went back up to a normal therapeutic level of 726 cells/mm^3^. 

## Discussion

In this report, we describe the clinical course of an HIV patient co-infected with COVID-19. During an HIV infection, there is depletion of the helper/inducer subset of T-lymphocytes, specifically the CD4+ marker, which results in immunosuppression [4]. The CD4+ lymphocyte cells are a marker of HIV-mediated cell damage, however, as we have noticed with COVID-19 patients, they can also be a guide to SARS-CoV-2-mediated cell damage [6]. In a previous study, it was highlighted that CD4+ cell cytopenia and immune dysregulation can be caused by the SARS-CoV-2 virus, and the investigators found that these patients were more likely to develop severe respiratory failure [7]. 

Upon hospital admission, the patient presented with an initial CD4+ count of 507 cells/mm³. As the infection progressed, we noticed a decreasing trend in his CD4+ cell count, dropping to a level of 102 cells/mm³. In this case of simultaneous COVID-19 and HIV infection, a significant decline of CD4+ levels, mimicking an AIDS clinical setting. Similarly, there have been recent studies that demonstrate the declining lymphocyte levels in patients with this coinfection. In a study of 292 patients, depleted levels of CD4+ lymphocytes offered insight on the prognosis of mortality based on the course or severity of the disease [8]. 

Simultaneous HIV and COVID-19 placed the patient at a higher risk of mortality. However, in this case the patient was discharged home without any complications. We hypothesize that this could be explained by the patient’s longstanding HIV diagnosis of 26 years, allowing him the appropriate time to achieve HIV viral suppression with antiviral therapy. It has been noted that patients living with HIV (PLWH) and taking antiviral therapy could have immunomodulatory effects by decreasing the production of inflammatory cytokines [9]. This in turn could potentially help attenuate the cytokine storm that is classically seen in severe COVID-19 cases by aiding and boosting their suppressed immune system [10]. 

The risk factor severity for each person with HIV could also contribute to increased immune response and mortality. This includes those with age greater than 65 years, hypertension, diabetes mellitus, chronic obstructive pulmonary disease, and kidney disease [9]. Our patient had hypertension and end-stage renal disease, which could also explain why despite having an undetectable viral load before admission, he developed a significant decline in CD4+ count when co-infected.

Conversely, in one retrospective study of 21 COVID-19 patients with HIV, it was noted that outcomes were worse because they required intensive care unit (ICU) care compared to the patients who did not have HIV [11]. This suggests that there may be an increase in the mortality rate among coinfected individuals, given the immunosuppressive nature of both of these viruses. Moreover, in another study that analyzed 88 coinfected patients, there was no significant difference found between the mortality rate of coinfected patients and non-coinfected patients [12]. Since the relationship between HIV and SARS-CoV-2 is not clear, further investigation is needed to determine if PLWH and COVID-19 are at greater risk for mortality. 

## Conclusions

There is still not enough information regarding the outcome of a patient who has co-infection with HIV and COVID-19. In our case, although the levels of CD4+ lymphocytes were declining, it was not associated with mortality. We also noticed that while these levels were declining throughout his hospital stay, once his COVID-19 infection was resolved, the levels of CD4+ lymphocytes also improved. We feel that a study in the population with HIV and COVID-19 co-infection is warranted to better understand the pathophysiology to help manage our patients more effectively.
